# The role of contaminated eggshells used in poultry feed in a diffuse nationwide outbreak of *Salmonella* Enteritidis, the Netherlands, 2023 to 2025

**DOI:** 10.2807/1560-7917.ES.2026.31.9.2500603

**Published:** 2026-03-05

**Authors:** Dana LL Adriaansens, Oda E van den Berg, Maren I Lanzl, Coen van der Weijden, Mark van Dommelen, Karin Nagel, Jenny Batstra-Blokpoel, Diederik AH Brandwagt, Kim van der Zwaluw, Kirsten Mooijman, Angela HAM van Hoek, Joline Mans-Poulie, Maaike van den Beld, Greetje Castelijn, Miriam Koene, Ife A Slegers-Fitz-James, Eelco Franz, Roan Pijnacker

**Affiliations:** 1 Centre for Infectious Disease Control, National Institute for Public Health and the Environment (RIVM), Bilthoven, the Netherlands; 2 Netherlands Food and Consumer Product Safety Authority (NVWA), Utrecht, the Netherlands; 3 The Netherlands Controlling Authority for Dairy and Eggs (COKZ), Leusden, the Netherlands; 4 Wageningen Food Safety Research (WFSR), Wageningen, the Netherlands; 5 Wageningen Bioveterinary Research (WBVR), Lelystad, the Netherlands

**Keywords:** *Salmonella* Enteritidis, Eggs, Poultry feed, Outbreak, The Netherlands, Whole genome sequencing

## Abstract

We describe a large and prolonged outbreak of *Salmonella* Enteritidis in the Netherlands. Between June 2023 and September 2025, we identified 227 outbreak cases (110 males, 114 females, three with missing information of sex; median age 43 years). Outbreak cases were individuals whose isolates belonged to the outbreak cluster based on whole genome sequencing (WGS) using single-linkage clustering with a threshold of ≤ 5 allelic differences, since June 2023. A case–control study focussing on egg consumption was conducted, alongside trace-back and trace-forward investigations. Findings of the case–control study confirmed the existence of two WGS subclusters: subcluster A linked to barn eggs (adjusted odds ratio (aOR) = 5.8; 95% confidence interval (CI): 2.11–15.99) and subcluster B linked to organic eggs (aOR = 63.6; 95% CI: 6.04–670.55). Isolates from 14 laying hen farms and eggshells were linked to the outbreak, suggesting the outbreak had multiple sources. Inadequate processing of contaminated eggshells before their use in poultry feed was most probably contributing to the spread and length of the outbreak. Measures to improve raw material control for animal feed were implemented, contributing to a decline in case numbers. However, since the outbreak likely had multiple sources, new cases continue to be detected, especially in subcluster B.

**Figure:**
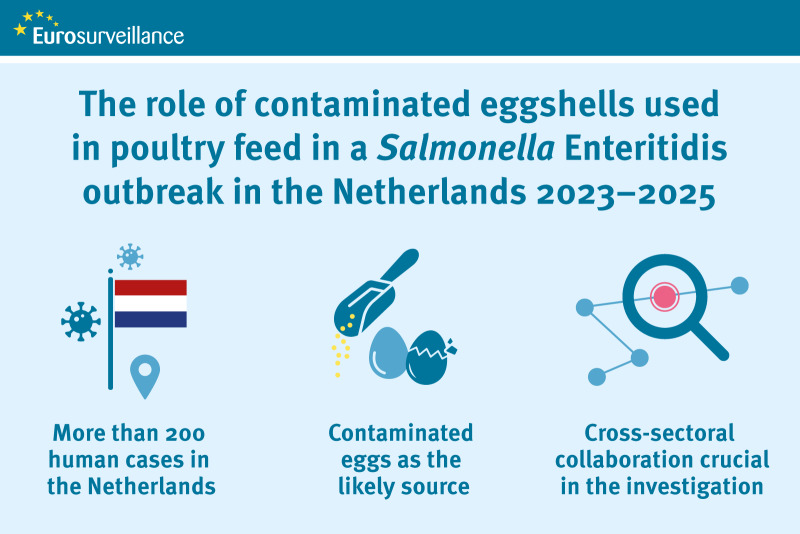

**Key public health message**

**What did you want to address in this study and why?**

*Salmonella* Enteritidis (SE) is a bacterium that is a major cause of diarrhoeal disease in humans in the Netherlands. Infection with SE is often linked to consumption of eggs and other poultry products. In July 2023, we detected nine patients with SE and aimed to identify the source(s) of this outbreak to prevent additional cases.

**What have we learnt from this study?**

We found that contaminated eggshells used in poultry feed contributed to the spread of the outbreak. This outbreak highlights the importance of pathogen elimination in feed production. Importantly, a voluntarily shared isolate originating from eggshells provided the key link in the investigation, underlining the value of data sharing across the human and animal health sectors.

**What are the implications of your findings for public health?**

Our findings highlight the need for robust controls and monitoring in animal feed production to prevent *Salmonella* contamination. Data sharing between public health, food safety and animal health sectors proved crucial in this investigation. Nevertheless, more frequent testing and timely sharing of isolates would aid future outbreak investigations and thereby benefit public health outcomes.

## Background

*Salmonella enterica* subspecies *enterica* serovar Enteritidis (SE) is a zoonotic pathogen and the most frequently reported serovar responsible for human salmonellosis in the Netherlands, with diarrhoea as the most common symptom [1]. This serovar is particularly associated with poultry as its reservoir and human infections are primarily associated with consumption of eggs [2]. Effective *Salmonella* control on poultry farms is based on biosafety measures and vaccination to prevent the introduction of *Salmonella* onto a farm [3]. The incidence of human SE infections in Europe declined significantly after the implementation of harmonised *Salmonella* control programmes in the European Union (EU) [4]. However, in recent years, the SE incidence has stabilised or even increased in some EU countries [2,5].

## Outbreak detection

In July 2023, a cluster of nine patients with SE was detected through the national genomic surveillance of the National Institute for Public Health and the Environment (RIVM). Five isolates from two different laying hen farms were genetically related to the cluster. An outbreak investigation was started to identify and eliminate the source of the infection. By October 2023, the number of cases had increased to 91, and based on whole genome sequencing (WGS) data and epidemiological questionnaire results, two distinct but genetically related subclusters were recognised. Here we describe the largest recorded SE outbreak to date in the Netherlands, which was investigated through cross-sectoral collaboration between RIVM and the competent authorities for animal health and food safety. Aspects of this outbreak have been briefly reported in a Eurosurveillance rapid communication [6].

## Methods

### *Salmonella* surveillance systems

In the Netherlands, salmonellosis in humans is not notifiable. National salmonellosis surveillance relies on voluntary laboratory reporting where clinical microbiology laboratories are requested to send culture-confirmed isolates to the RIVM for characterisation and typing with WGS. These isolates are accompanied by a minimal set of patient data, including sampling date, date of birth, sex, specimen type (e.g. blood, faeces), postal code, city of residence and travel history when available.

*Salmonella* is monitored through national surveillance programmes of the Netherlands Food and Consumer Product Safety Authority (NVWA), including programmes for feed monitoring [7] and the verification of correct handling of contaminated eggs by packing centres [8]. *Salmonella* in laying hens is monitored in accordance with EU regulations (EC) 2160/2003 [9] and 517/2011 [10]. Adult laying hen flocks are sampled every 15 weeks during the laying period, starting from the age of 24 weeks (± 2 weeks). These samplings are carried out by Food Business Operators (FBO). Additionally, adult laying hen flocks are sampled by veterinary personnel 3 weeks before slaughter. If *Salmonella* spp. is confirmed, restrictive measures are taken, that apply to all eggs produced from the sampling date onwards, including those at the packing centre. After detection of *Salmonella* spp., serotyping according to the White-Kauffmann-Le Minor scheme [11] is performed by approved laboratories. If SE or *Salmonella* Typhimurium (ST) is found, the flock is declared infected according to the legislative criteria. If another serovar is detected, the suspicion is lifted and the restrictions are removed. There is a reporting obligation for both FBOs and laboratories concerning *Salmonella* spp. and the relevant serovars in poultry samples. All data must be documented and stored in a designated database which is commissioned by the Ministry of Agriculture, Fisheries, Food Security and Nature. Poultry farmers have access to their individual data and are responsible for its accuracy. Regulators have full access to all data.

### Data sources

The surveillance system for human salmonellosis described above served as the main data source during this outbreak. In addition, on 19 September 2023, Dutch clinical microbiology laboratories, that were not part of the regular surveillance network, were asked to prospectively submit *Salmonella* group D isolates, which includes SE, to help provide a more comprehensive overview of the outbreak.

Data on SE in laying hen flocks were obtained through the previously described monitoring programmes. In addition, between 1 and 26 October 2023, the poultry sector decided that all laying hen farms had to perform additional sampling for *Salmonella* (SE and ST) in all their flocks. The additional sampling may have resulted in earlier detection of *Salmonella* in some flocks.

### Outbreak case definition

An outbreak case was defined as a person reported since June 2023 with a laboratory-confirmed SE isolate showing a core-genome multilocus sequence typing (cgMLST) allelic difference (AD) ≤ 5 to another isolate belonging to one of the two outbreak subclusters (A and B). Historical cases were defined as persons reported before June 2023 with a laboratory-confirmed SE isolate showing a cgMLST AD ≤ 5 to an isolate in subcluster A or B.

### Epidemiological investigation

As part of the epidemiological investigation, a questionnaire was administered focussing on egg and poultry meat consumption in the week before symptom onset. The questionnaire included details of the retailer from which the products were purchased: the type of eggs, whether eggs were still available, either to record the egg code or to collect them for testing. Egg codes, numbers stamped on eggs providing information about the egg’s origin (country, laying hen farm and house) and type of farming system (e.g. organic, free range), were recorded, to establish a direct link between patients and layer houses. The symptom onset date was estimated when missing, using the average time (12 days) between the arrival of the isolate at the RIVM laboratory and the reported symptom onset date for cases with available data. Case interviews were performed by the Municipal Health Service (MHS) of the case’s residential location. The MHS staff received an online questionnaire from the RIVM and either filled in over the phone together with the patient or forwarded to the case to complete. Relevant questionnaire data were shared with the NVWA to enable trace-back investigations of suspected food items.

On 14 September 2023, a case–control study was initiated. Three rounds of control selection were conducted between September and November 2023, and the analysis was carried out in December 2023. In the first round, four controls were selected per case. In the second and third rounds, six controls were selected per case. All controls were randomly sampled from the population registry and frequency matched by age, sex and municipality. Controls were invited by mail to complete an online questionnaire.

Data were analysed using logistic regression to calculate adjusted odds ratios (aOR) and 95% confidence intervals (95% CI). The model was adjusted for age and sex. Variables with a p value < 0.1 were included in a multivariable model, where only variables with a p value < 0.05 were retained after backward stepwise selection. The analyses were performed in RStudio version 4.4.3 (https://www.r-project.org/), with the packages readxl [12], tidyverse [13], stringi [14] and rio [15]. Generalised linear models were constructed using the glm() function.

### Sequencing and genomic comparison

All isolates of SE (human and non-human) sent to the RIVM were subjected to WGS using the Illumina NextSeq platform (Illumina, San Diego, the United States (US)) [16]. These included isolates submitted by the NVWA and other non-human isolates sent to RIVM from commercial companies. All isolates are stored for a 10-year period. Isolates from raw material for feed and environmental swabs were sequenced by Wageningen Food Safety Research (WFSR) on behalf of the NVWA, using WGS on the Illumina NovaSeq platform [17]. The raw sequence data files are routinely exchanged between both institutes with a 4–6 week interval. At the RIVM, sequences of human and non-human isolates were processed through an in-house developed pipeline “Juno assembly” consisting of read trimming followed by de novo assembly, including quality control of raw reads, trimmed reads and assemblies [18]. The output was processed using an in-house developed “Juno typing” pipeline which includes in silico *Salmonella* serotyping with SeqSero2 and 7-locus MLST analysis [19,20]. De novo assemblies were imported in Ridom Seqsphere+ [21] to determine allelic profiles using the Enterobase *S*. *enterica* cgMLST template scheme (3,002 targets) (https://enterobase.warwick.ac.uk/). Distance matrices were then calculated, with pairwise missing loci being ignored. Clusters were defined using a hierarchical agglomerative clustering approach with single linkage, applying a cut-off  ≤ 5 AD.

Furthermore, EpiPulse, the surveillance portal for European public health authorities (https://www.ecdc.europa.eu/en/publications-data/epipulse-european-surveillance-portal-infectious-diseases) was used to share FASTA files of representative outbreak strain sequences with other EU countries to investigate whether similar sequences had been identified in their national surveillance systems.

### Environmental and trace-back investigations

Trace-back and trace-forward investigations were performed by the NVWA and the Netherlands Controlling Authority for Dairy and Eggs (COKZ) to identify possible routes of infection, based on non-human isolates identified as part of the outbreak cluster through WGS data analysis. All related isolates, provided they were not submitted anonymously (i.e. without traceability to their origin), were included in the tracing investigations. Additionally, trace-back analyses were conducted using information from case questionnaires, including egg codes. As a result, egg samples were collected from patients’ homes and further boot swab samples were taken on several laying hen farms associated with the egg codes. Furthermore, additional environmental swabs were taken at an egg packing centre after a routine sample from its production environment was genetically linked to the outbreak cluster.

## Results

### Descriptive epidemiology

**Figure 1:**
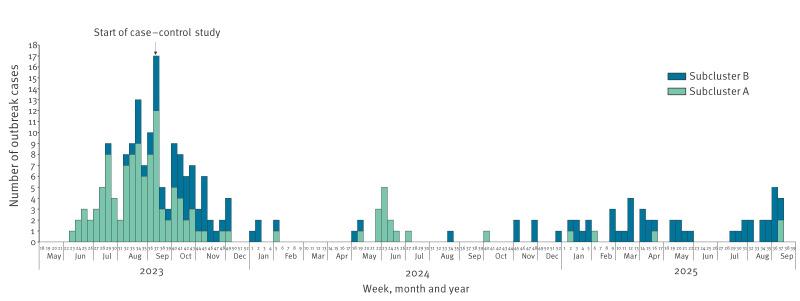
Epidemiological curve of *Salmonella* Enteritidis outbreak cases, by symptom onset date, the Netherlands, May 2023–September 2025 (n = 227)^a^

We identified 227 cases between June 2023 and September 2025. Before June 2023, 28 historical cases were identified originating from the period between November 2018 and January 2023. After June 2023, the number of cases rapidly increased, which prompted the start of the outbreak investigation (Figure 1). The outbreak peaked in week 37 (September 2023), after which the case numbers gradually decreased until the end of the year. After almost 3 months without cases, several new cases were identified between May and July 2024. In 2025, case numbers began to increase again, with 44 of 49 cases belonging to subcluster B. By September 2025, 227 cases (excluding the historical cases before June 2023) were identified of which 151 in 2023, 27 in 2024 and 49 in 2025. Of these, 110 were males, 114 females and three without information on sex. Cases ranged in age from 1 to 98 years (25^th^ percentile: 20; 50^th^ percentile: 43; 75^th^ percentile: 67). *Salmonella* Enteritidis was detected in stool samples of 205 cases, in blood samples of 12 and in samples from other or unknown body sites of 10.

RIVM: the National Institute for Public Health and the Environment.

^a^ If the date of symptom onset was missing, it was estimated by subtracting 12 days from the arrival date of the isolate at the RIVM laboratory.

Subcluster A consisted of 123 cases (65 males, 56 females, two with missing information on sex; age range: 1–98 years; median age: 40 years) and subcluster B of 104 cases (58 females, 45 males, one with missing information on sex; age range: 1–87 years; median age: 45 years). The first case in subcluster A developed symptoms in week 23 (June 2023), and the first case in subcluster B in week 29 (July 2023).

### Microbiological investigation and whole genome sequencing

**Figure 2:**
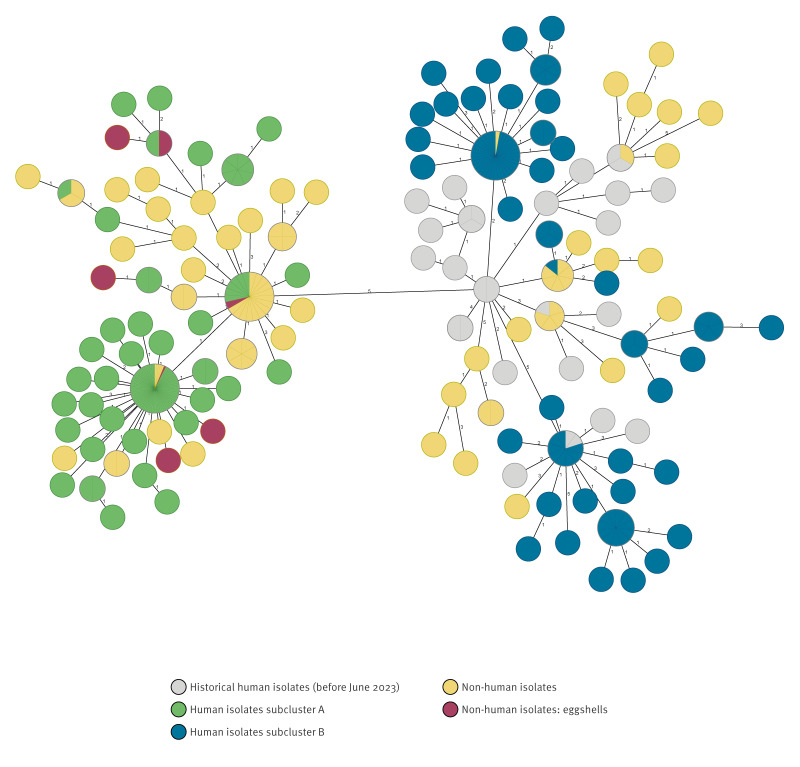
Minimum spanning tree of *Salmonella* Enteritidis sequences from samples from humans and non-human sources, the Netherlands, 2018–2025 (n = 345)^a^

^a^ Cluster cut-off  ≤ 5 AD using single-linkage clustering.

In September 2023, WGS clustering revealed that the outbreak consisted of two closely related subclusters, A and B. The distance between subclusters A and B, determined based on the distance between the nearest outbreak isolates of each subcluster, was 6 AD. All non-human SE isolates, included as outbreak isolates, were from samples taken between October 2021 and February 2025. The maximum distance between outbreak isolates in subcluster A was 9 AD and 20 AD in subcluster B. All isolates (outbreak and historical) exhibited a maximum of ≤ 5 AD, when using single-linkage clustering (Figure 2). In total, 59 non-human isolates (29 boot swabs, 14 other poultry, 7 dried eggshells, 6 poultry faeces, 2 environmental samples from a packing centre and 1 unknown) were part of subcluster A and 31 (22 boot swabs, 8 other poultry and 1 chicken neck skin) of subcluster B. Known duplicate isolates were excluded. Other poultry refers to isolates submitted anonymously, with only information of the animal species included.

The EpiPulse (event ID: 2023-FWD-00047) inquiry suggested that the outbreak was confined to the Netherlands, as only one case in another EU country was identified (case had no travel history). Furthermore, two historical EpiPulse sequences from 2021 were related to subcluster B. Direct inquiry with colleagues from another EU country identified nine cases closely related to the outbreak cluster, one of whom reported travel to the Netherlands. A possible connection was also identified between some of these cases and eggs that may have originated from the Netherlands.

### Case–control study

In the case–control study, conducted between September and December 2023, we compared 90 cases (subcluster A: n = 64; subcluster B: n = 26) with 94 controls (subcluster A: n = 72; subcluster B: n = 22). The results suggested that subcluster A and B had partly different epidemiological characteristics (Table). In subcluster A, cases were significantly more likely to have purchased eggs at a supermarket (aOR = 6.1), consumed barn eggs (aOR = 5.8) or consumed soft-boiled eggs (aOR = 2.1) than the controls. Furthermore, cases in subcluster A were less likely to have purchased eggs at a farm (aOR = 0.2) or consumed organic eggs (aOR = 0.2) than the controls. Subcluster B cases were significantly associated with consumption of organic eggs (aOR = 63.6) and soft-boiled eggs (aOR = 4.6). Cases of subcluster B consumed barn eggs (aOR = 0.1) less often than controls. Most cases in subcluster A (60/64; 94%) and B (21/26; 81%) purchased their eggs at supermarkets.

**Table:** Summary of significant case–control study outcomes in an investigation of an outbreak with *Salmonella* Enteritidis, the Netherlands, June 2023–December 2023 (n = 90)

Characteristic	Cases (n = 90)			Controls (n = 94)			aOR	95% CI	p value
	Exposed (n)	Total (n)	%	Exposed (n)	Total (n)	%			
Subcluster A (cases: n = 64; controls: n = 72)									
Meat purchase location									
Meat butcher	2	63	3	20	72	28	0.1	0.02–0.34	0.001
Groceries purchase location									
Supermarket A	23	64	36	44	72	61	0.3	0.16–0.68	0.003
Supermarket B	38	64	59	24	72	33	2.8	1.39–5.79	0.004
Egg purchase location									
Supermarket	60	64	94	51	72	71	6.1	1.97–19.16	0.002
Farm	3	64	5	17	72	24	0.2	0.04–0.55	0.004
Type of egg consumed									
Barn egg	31	38	82	21	49	43	5.8	2.11–15.99	0.001
Organic egg	2	38	5	12	49	24	0.2	0.03–0.81	0.026
Other	5	38	13	1	49	2	9.6	1.02–89.90	0.048
Egg preparation method									
Other	21	61	34	39	70	56	0.4	0.20–0.82	0.013
Soft-boiled egg	34	60	57	28	70	40	2.1	1.04–4.38	0.039
Subcluster B (cases: n = 26; controls: n = 22)									
Type of egg consumed									
Organic egg	19	21	90	3	15	20	63.6	6.04–670.55	0.001
Barn egg	3	21	14	11	15	73	0.1	0.01–0.34	0.001
Egg preparation method									
Soft-boiled egg	16	25	64	7	22	32	4.6	1.25–16.97	0.022

aOR: adjusted odds ratio; CI: confidence interval.

### Tracing and environmental investigations

Trace-back investigation based on consumer egg purchase information proved to be challenging because it was not possible to identify the specific layer house from which the eggs had originated. This was in part due to the lack of egg codes retrieved from patients, which resulted from time delays between egg consumption and case interviews. Additionally, tracing eggs purchased at supermarkets was challenging at the brand level, as egg boxes from the same brand could contain eggs from different layer houses or even from multiple laying hen farms.

**Figure 3:**
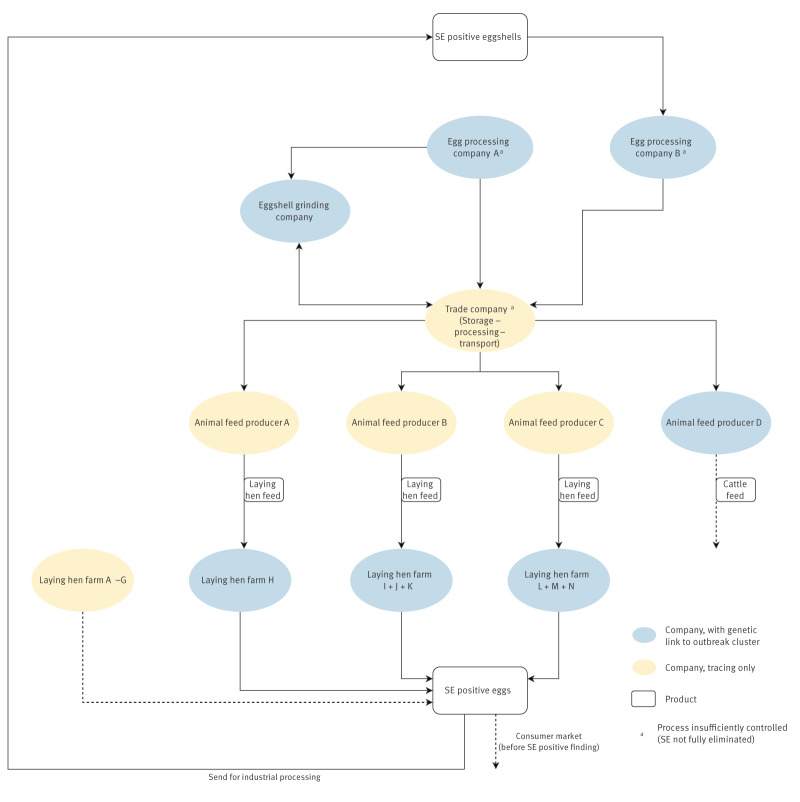
Visualisation of tracing investigation of an outbreak with *Salmonella* Enteritidis, the Netherlands, June 2023–September 2025

Isolates from 14 laying hen farms and one packing centre were genetically linked to the outbreak, indicating that multiple potential sources contributed to the outbreak. All eggs from laying hen flocks tested positive for SE, including those associated with this outbreak, had to be sent for industrial processing during which *Salmonella* must be eliminated. Thus, eggs from these layer houses could no longer contribute to the outbreak, except for eggs already placed on the consumer market before the finding of SE. With the source of the outbreak still unclear, progress in the investigation stalled. However, a breakthrough was achieved in week 41 (October 2023), when an isolate obtained from dried eggshells collected as part of an internal monitoring programme of an animal feed producer was genetically linked to subcluster A. The batch of eggshells related to this isolate was used as raw material for cattle feed. This discovery shifted the investigation towards feed as a potential source. Further tracing by the NVWA and COKZ revealed that eggshells were insufficiently processed to eliminate *Salmonella* before their use as a calcium source in poultry feed. Inadequate processing could have resulted from insufficient heat treatment, or from recontamination during transport or storage. This was substantiated by detection of SE on eggshells from two egg processing facilities, one eggshell grinding company and one animal feed manufacturer. Isolates from these seven samples were genetically linked to subcluster A (three other animal feed manufacturers were linked to the outbreak based on tracing only) (Figure 3). These findings revealed the most likely transmission chain, in which eggshells from SE-positive layer houses were used in poultry feed without adequate treatment to eliminate *Salmonella.*

SE: *Salmonella* Enteritidis.

## Outbreak control measures

After discovering that eggshells had been inadequately processed to eliminate *Salmonella* before being used in animal feed, control measures were taken to prevent further transmission. Monitoring of the treatment process was intensified at the implicated companies, followed by subsequent audits, particularly on hygiene, monthly monitoring of the microbiological quality of the products, traceability and process parameters and their monitoring. Later, companies that were not initially identified through tracing were also subjected to additional audits by the NVWA. These audits were conducted at all processors of animal by-products derived from eggs and at companies supplying organic fertilisers and soil improvers. Following the audits, all egg processing and grinding companies self-revalidated and further automated their process parameters, with some even investing in new processing installations. Following the introduction of the control measures, which are still in effect, case numbers in both subclusters started to decrease.

General and outbreak specific information about *Salmonella* was published on the RIVM website in November 2023, with details on the suspected source added in January 2024 [22]. Public health professionals were informed through the weekly early warning email on infectious diseases, and medical microbiologists were notified through the national alert system for laboratories, known as LabInf@ct.

## Discussion

With 227 outbreak and 28 historical cases, this was the largest SE outbreak recorded in the Netherlands to date. However, this is an underestimation of the outbreak size, as it is estimated that for every reported case with an isolate, there are 26 additional cases [23]. Epidemiological investigations, including WGS clustering, identified consumption of eggs from Dutch laying hen farms as the most probable source of the outbreak. However, tracing the source of the outbreak was challenging as various types of eggs from multiple laying hen farms in the Netherlands were potential sources of the infection. Therefore, the investigation shifted its focus to identifying the source of the contamination further upstream in the egg production chain. The breakthrough was the identification of an isolate from dried eggshells that matched subcluster A. Dried eggshells are used as a calcium source in laying hen feed [24]. However, eggshells can be contaminated with microbial pathogens and must therefore undergo proper treatment to eliminate these microorganisms [25]. Relevant parameters for effective treatment include time, temperature, particle size and pressure. In this outbreak, the processing proved insufficient either due to inadequate heat treatment or recontamination. As a result, eggshells contaminated with SE were used in the production of poultry feed, which most likely contributed to the prolonged and diffuse nature of the outbreak. Several factors remain unknown, including how the pathogen was initially introduced into the egg production chain and the routes of transmission to farms where SE was detected but with no traceable link to the implicated feed. In particular, the source of subcluster B remains unclear, as none of the eggshell isolates closely matched this subcluster. However, cases in both subclusters decreased after implementation of the interventions.

While these control measures were important, they were likely not the sole factor contributing to the reduction in case numbers. The situation also raised awareness within the sector, particularly about the risks associated with inadequately treated eggshells used as raw material for feed. This increased awareness prompted additional proactive measures by the farmers themselves, such as avoiding feed from the implicated manufacturers and improving their own biosafety practices. Greater emphasis was placed on hygiene and traceability among chain partners, including transport, grinding installations and storage facilities. These efforts likely contributed to the initial decline in case numbers. However, the increase in cases belonging to subcluster B in 2025 suggests that the outbreak had multiple sources.

This outbreak emphasised the importance of cross-sectoral information sharing (human and animal/environmental), as the isolate originating from dried eggshells voluntarily submitted by an animal feed producer was a key factor in the investigation. Nevertheless, future outbreak investigations would benefit from more frequent testing and timely sharing of isolates by the primary sector. For instance, isolates from certain related laying hen farms sampled in 2023 were retrospectively sequenced in 2024. More real-time data sharing might have enabled earlier identification of these laying hen farms as part of the outbreak. The Dutch competent authorities are exploring options to implement more frequent routine testing and sharing of isolates and/or WGS data. Additionally, as a consequence of this outbreak, the laying hen sector has increased testing frequency of laying hens older than 65 weeks, reducing the interval from every 15 weeks to every 8 weeks as of January 2024 [26]. Due to the ongoing increase in SE cases in the Netherlands [6], the sector further strengthened these measures in October 2025: younger laying hens are now tested every 8 weeks instead of every 15 weeks and laying hens older than 65 weeks are tested every 4 weeks [27].

## Conclusion

While animal feed is recognised as a potential risk factor for introducing *Salmonella* on farms, this outbreak shows the important role of pathogen elimination measures in feed production. Mandated control measures, combined with increased awareness of the risks associated with contaminated eggshells used as raw material for feed in the laying hen sector likely contributed to the decline in patient numbers. This highlights the importance of both regulatory actions and proactive efforts within the sector. Investigating the egg production chain proved to be complex. However, the use of WGS and isolate/data sharing enhanced the outbreak investigation, underscoring the value of advanced molecular tools and cross-sectoral collaboration in improving food safety and public health outcomes.

## Data Availability

Sequencing data of human isolates are available from the corresponding author upon reasonable request. Request for sequencing data of non-human isolates will be considered on a case-by-case basis.
